# Integration of AI diagnostic tools into clinical practice for Alzheimer’s disease: barriers and solutions

**DOI:** 10.1097/MS9.0000000000004505

**Published:** 2025-12-18

**Authors:** Muhammad Umer Suleman, Muhammad Mursaleen, Umer Khalil, Shahbaz Azam Khan, Abdul Saboor, Muhammad Ali Hussnain, Mohammad Zahir, Usman Ayaz Khan, Dawood Ur Rehman, Syeda Nafisa Tabassum

**Affiliations:** a Department of Internal Medicine, Ayub Medical College, Abbottabad, Pakistan; b Department of Microbiology, Bangladesh Rural Advancement Committee: BRAC, Dhaka, Bangladesh

**Keywords:** Alzheimer disease, artificial intelligence, biological, biomarkers, cognitive dysfunction, diagnostic imaging, machine learning, wearable electronic devices

## Abstract

Alzheimer’s disease is a progressive neurodegenerative disorder that affects millions of people worldwide and remains difficult to diagnose in its earliest stages. This narrative review examines developments in artificial intelligence diagnostic tools designed to support clinicians in the detection of Alzheimer’s disease. It evaluates systems that analyze brain imaging scans, genetic information, and cognitive assessments, as well as emerging approaches that monitor speech patterns and data from wearable devices. The review identifies six challenges to clinical adoption: limited and unrepresentative data sets; limited transparency of algorithmic decisions; disruption of established clinical workflows; unclear regulatory frameworks; high implementation costs and infrastructure demands; and the potential to widen health disparities. To address these issues, we propose the creation of large collaborative data repositories, the advancement of transparent model interpretation methods, comprehensive clinician education programs, the establishment of clear regulatory pathways, and strategic investment in scalable infrastructure. By confronting these technical, human, and system-level challenges through coordinated efforts, artificial intelligence diagnostic tools can be incorporated into Alzheimer’s disease care to enhance early diagnosis and improve patient outcomes across diverse healthcare settings.

## Introduction

Alzheimer’s disease (AD) is a progressive neurodegenerative disorder and the most common cause of dementia, accounting for roughly 60–70% of dementia cases^[1,2]^. It is a leading cause of death and disability worldwide^[3]^. In 2019, an estimated 57.4 million people had dementia globally, and that number is projected to rise to approximately 152.8 million by 2050^[4]^. The earliest clinical features of AD typically involve memory and language impairments (reflecting damage in brain regions responsible for these functions), although neuropathological changes begin decades before symptoms appear^[5–8]^. This long asymptomatic period underscores the importance of timely and accurate diagnosis so that patients can be managed and interventions initiated as early as possible^[9]^. Despite its importance, early and accurate diagnosis of AD remains challenging due to overlapping symptoms and the need for expensive tests^[10–14]^. Artificial intelligence diagnostic tools [e.g., machine learning (ML) algorithms applied to imaging, biomarkers, genetics, and digital data] promise to augment clinicians by detecting subtle patterns and biomarkers of AD^[15–17]^. In principle, AI can integrate heterogeneous patient data [from magnetic resonance imaging (MRI) and positron emission tomography (PET) scans to genetic profiles and cognitive tests] to improve diagnostic accuracy and speed^[18,19,20]^. For example, large research datasets like the UK Biobank (500 000 individuals with imaging and genomic data) are being used to train AI models on AD-related features^[21]^.

**HIGHLIGHTS**

- AI tools enhance early Alzheimer’s diagnosis through data integration.
- Major barriers include bias, workflow disruption, and poor interpretability.
- Federated learning helps improve model accuracy across diverse populations.
- Clinician trust and training are essential for successful AI adoption.
- Global equity requires low-cost tools and inclusive data collaboration.

This narrative review explores recent advancements in AI-based diagnostic tools for AD, with a focus on both high-income and low-income settings. We examine the current applications of AI in AD diagnosis, including neuroimaging analysis, digital cognitive assessments, and emerging technologies like speech analysis and wearable data. The review identifies key barriers hindering the clinical integration of these tools, including data limitations, algorithmic biases, regulatory challenges, and a lack of clinician trust and training. To address these challenges, we propose a range of strategies, emphasizing the need for data collaboration, clinician education, and the development of interpretable AI models to ensure trust and transparency. The goal of this review is to provide a comprehensive synthesis of the current landscape, highlighting the potential of AI in AD care while also outlining the steps necessary for its broader adoption in both developed and resource-limited settings. In the preparation of this article, AI-assisted tools were used in accordance with the TITAN Guidelines 2025^[22]^.

## Methodology

The methodology for this narrative review was designed to investigate the integration of artificial intelligence diagnostic tools into clinical practice for AD. A comprehensive literature search was conducted using major databases with a focus on publications between 2010 and 2025. Keywords such as “Alzheimer’s disease,” “AI,” “machine learning,” “diagnostic imaging,” and “biomarkers” were used to identify relevant studies, technical reports, and reviews. Studies that discussed AI tools specifically for AD diagnosis were included, particularly those addressing neuroimaging, genetic assessments, and cognitive testing. Articles were also included if they examined the barriers to clinical adoption of AI tools, such as data limitations and regulatory challenges. Publications reporting on the performance of AI diagnostic tools, including accuracy, sensitivity, specificity, and clinical validation, were prioritized.

Inclusion criteria were strictly defined to focus on studies discussing AI-based diagnostic tools in the context of AD, including clinical trials and development papers. Articles that did not provide sufficient data on AI tool performance or were unrelated to AD diagnosis were excluded. Non-English language studies were also excluded due to the inability to reliably review them. Following the search, a selection of relevant studies was reviewed in detail, extracting key data on the applications of AI tools, their performance in AD diagnosis, and the specific challenges reported by the authors.

The studies were analyzed qualitatively, and the information was synthesized into thematic areas such as AI applications, barriers to adoption, and solutions for integration. Particular attention was given to the technological, human, and systemic barriers preventing AI adoption in clinical settings. The review focused on emerging methods like explainable AI (XAI) and federated learning to address these challenges, emphasizing the need for transparent, interpretable models that can enhance clinician trust and improve integration into existing workflows. Key findings from the literature were compared and contrasted to identify trends and gaps in the current research on AI in AD diagnosis. This synthesis was presented in a narrative format, supported by tables summarizing the AI diagnostic tools discussed and their current status in clinical use.

## Current applications of AI in Alzheimer’s diagnosis

Artificial intelligence is increasingly being applied to Alzheimer’s diagnosis, leveraging various forms of clinical data, including neuroimaging, biomarkers, genetic profiles, and cognitive tests. Several AI tools are currently in development or early use, with promising results in enhancing diagnostic accuracy and efficiency. These tools use advanced ML algorithms to detect subtle patterns that may not be easily identified through conventional methods. Table 1 lists examples of AI-based diagnostic tools for AD that are in development or are in early use. These include imaging-analysis systems and novel sensing technologies. For instance, Mayo Clinic’s StateViewer is a fluorodeoxyglucose (FDG)-PET machine-learning tool that identifies nine dementia types (including AD) from a single brain scan. In trials, it achieved high performance [sensitivity ~0.89, area under the curve (AUC) 0.93] in multi-class dementia classification^[23]^. Similarly, AIRAscore (by AIRAmed) is a Food and Drug Administration (FDA)-cleared MRI-processing AI that automatically quantifies brain atrophy and other markers of AD; this tool can integrate seamlessly into radiology workflows, is relatively low-cost, and is quick to configure^[24]^. Other AI devices include NeuroQuant (Cortechs.ai), a fully automated MRI volumetry software that has FDA 510(k) clearance for labeling and quantifying brain structures (now upgraded to detect amyloid-related lesions in AD patients)^[25]^. Beyond imaging, emerging approaches are under study: for example, RetiSpec’s retinal hyperspectral imaging system (now partnered with Topcon) aims to detect AD-specific biomarkers (amyloid-β) via AI analysis of retinal scans^[26]^. These examples demonstrate that AI tools can not only leverage existing clinical data to enhance AD diagnosis but also highlight that most are still in research or early rollout stages (Table 1).

**Table 1:** Summary of example AI diagnostic tools for Alzheimer’s disease

Tool	Developer/maker	Data modality	Purpose/use case	Status	Category
StateViewer	Mayo Clinic^[27]^	FDG-PET brain scan	Automated classification of neurodegenerative diseases (including AD)	Research prototype; shown 0.93 AUC in multi-class neurodegenerative diseases diagnosis; under further clinical evaluation^[10]^.	Experimental
AIRAscore	AIRAmed^[24]^	MRI brain volumetry	Quantification of cortical and subcortical volumes, detecting AD atrophy	FDA 510(k) cleared (2023) for MRI volumetry and early AD detection; CE-marked in the EU. Affordable, quick integration^[11]^.	FDA/CE-cleared
NeuroQuant	Cortechs.ai^[25]^	MRI brain volumetry	Automated labeling and volumetry of segmentable brain structures	Commercially available since 2007; FDA cleared for automatic MRI analysis; latest v5.0 cleared (2024) for ARIA lesion quantification^[12]^.	FDA/CE-cleared
RetiSpec & Topcon	RetiSpec & Topcon Healthcare^[26]^	Hyperspectral retinal imaging	Non-invasive screening for AD biomarkers (retinal amyloid)	Research/clinical validation stage; planned integration into ophthalmic workflow via Topcon Harmony platform^[13]^.	Experimental
Others (examples)	–	Speech/language, gait, EEG	Exploratory tools analyzing voice, movement, or EEG patterns linked to cognitive decline	Under active research; no widely deployed products yet.	

ARIA, amyloid-related imaging abnormalities.

## Barriers to clinical integration

Despite promising performance, multiple barriers hinder the routine use of AI diagnostics for AD. Key challenges include the following:

**Data limitations and bias**: Robust AI requires large, diverse labeled datasets. However, AD datasets are often limited and non-representative. Many models are trained on data from a single population (e.g., North American or European cohorts), so they underperform on others. A systematic review found that AI models developed in the USA were 12–25% less accurate when tested on Asian and African populations^[21]^. This generalizability gap arises from genetic, environmental, and imaging differences. Moreover, rare AD subtypes or early-stage cases are underrepresented, limiting model sensitivity. Relatedly, federated learning has been proposed to mitigate data sharing constraints, and one study reported that it improved disease detection AUC by 11% in worldwide cohorts, but broad data collaboration is still lacking^[21]^.

**Lack of interpretability (trust**): Many high-performing AI algorithms (especially deep learning) operate as “black boxes.” Clinicians are hesitant to trust a diagnostic output if they cannot understand the rationale^[28]^. Indeed, fewer than 8% of FDA-cleared AI tools provide explanations of their decisions^[21]^. This opacity raises liability and ethical concerns. Radiologists and neurologists often demand transparent reasoning or visual heatmaps of abnormalities before accepting AI suggestions. Without interpretability, AI recommendations may face resistance and cannot easily fit into evidence-based workflows.

**Clinical workflow and acceptance**: Physicians and allied health providers must adopt and use the tools in practice. Studies in radiology show clinicians’ lack of knowledge and trust in AI, as well as fear of automation replacing their role, were major barriers^[29–32]^. Similarly, allied health professionals cite limited AI training and uncertainty about its impact on jobs as concerns^[33,34]^. Many doctors need education on AI capabilities and limitations; without this, they may ignore or override AI outputs. Also, integrating an AI tool often requires changes to established workflows (e.g., adding a step to upload scans to the AI software), which can be resisted if no clear benefit is shown.

**Regulatory and ethical issues**: AI diagnostic tools for AD often fall under medical device regulations. Clear approval pathways (e.g., FDA 510(k) and EU CE marking) are needed, but many algorithms are not yet classified or approved. Regulators are only now issuing guidance (e.g., FDA’s AI/ML Action Plan and draft frameworks for change management)^[35]^. The FDA, for instance, has recognized the need for a tailored approach to software as a medical device (SaMD) and issued the FDA AI/ML SaMD Action Plan, which provides guidance for the pre-market and post-market regulatory processes for AI and ML-based tools^[36]^. The plan emphasizes the development of a transparent and iterative process to ensure AI systems are safe and effective over time, addressing the dynamic nature of these technologies. Similarly, the EU AI Act, introduced by the European Commission, seeks to regulate high-risk AI systems, including those used in healthcare, by establishing requirements for transparency, accountability, and human oversight^[37]^. The Act classifies AI tools in healthcare as high-risk, meaning they must comply with strict regulations to ensure safety and reliability. These regulations address the need for continuous monitoring and validation to avoid misdiagnosis and safeguard patient well-being. Until standards for validation and monitoring are established, clinics may avoid unapproved AI. Ethical questions also arise: e.g., if an AI predicts cognitive decline, how is patient consent and counseling handled? Privacy is a concern when training on sensitive data (imaging, genetics). Healthcare workers have flagged risks of misdiagnosis and loss of confidentiality with AI screening^[38]^.

**Infrastructure and cost**: Practical deployment requires integration into existing IT systems [picture archiving and communication systems (PACS) and electronic health records (EHR)]; without standards-based hooks into those systems, an AI model cannot reliably receive images, return results, or fit into clinician workflows^[39]^. Many clinics lack the computing infrastructure or interoperability needed^[40]^. Smaller centers may not afford new AI subscriptions. While some tools (like AIRAscore) emphasize ease-of-use and low cost, many AI solutions involve cloud computing or specialized hardware^[24]^. Clinics also lack reimbursement models for AI diagnostics; without billing codes or proof of cost-effectiveness, hospitals are reluctant to pay for these tools^[41]^.

**Global and equity gaps**: AI adoption is highly uneven globally. High-income countries (HICs) lead research and have advanced digital infrastructure, whereas low- and middle-income countries (LMICs) face a “deployment paradox”^[42,43]^. In LICs, limited internet access, few trained professionals, and a lack of policy support hinder AI use^[42,43]^. Without targeted efforts, AI in AD could worsen health disparities; for example, models trained on a single dataset, often from HICs, may not generalize to other populations^[44,45]^. Developed nations must consider global equity by sharing knowledge and data.

The *summary of barriers* mentioned earlier is also illustrated in Table 2. Broadly, the challenges span technical, human, and systemic factors. Overcoming them requires multi-pronged solutions (next section).

**Table 2:** Key barriers to integrating AI diagnostics in clinical practice

Barrier category	Description/impact
Data & algorithmic	Small or biased datasets (e.g., AD neuroimaging initiative’s mostly white elderly cohort) lead to poor generalizability^[21]^. Lack of interoperability standards (imaging formats, labels) further hampers development. Limited data sharing (due to privacy laws) prevents building robust models, especially for underrepresented groups.
Interpretability/trust	“Black box” outputs without explanations undermine clinician trust^[21,46]^. Few tools offer transparent reasoning. Ethical/legal accountability for AI decisions is unclear, making regulators and physicians wary.
Clinical workflow	Clinicians may lack AI literacy; many have reservations or feel threatened^[29,33]^. New tools disrupt established processes; limited time and training means few will adopt unless efficiency gains are obvious. Poor user interface or integration (e.g., requiring separate apps) lowers uptake.
Regulatory & ethics	Inconsistent or nascent regulations for AI/ML medical devices cause uncertainty. Ethical issues (consent for AI analysis, data privacy) must be addressed. Without approved guidelines and post-market surveillance, hospitals hesitate to deploy AI.
Economic & infrastructure	Upfront costs (software, hardware) and unclear reimbursement impede adoption. Many AI tools need advanced computing or cloud access, which some clinics lack. Cost–benefit is not yet proven for payers. Integrating AI outputs into EHR/PACS is technically challenging.
Global disparities	Low-resource settings lack digital infrastructure, trained personnel, and local data^[42]^. AI solutions developed in HICs may not suit developing countries. Without inclusive policies and international support, a “digital divide” in AD diagnostics will grow.

## Solutions and strategies

A range of strategies has been proposed to address these barriers:

**Data collaboration and improved datasets**: Building diverse, large-scale datasets is crucial. Initiatives should pool data across centers and countries (e.g., extension of AD neuroimaging initiative to new regions) to cover different ethnicities and disease stages^[47–49]^. Privacy-preserving techniques like federated learning can allow model training on distributed data without sharing raw records^[21]^. Standardizing acquisition protocols and labels through international consortia improves algorithm robustness and reproducibility; data augmentation and synthetic-image methods can increase sample diversity but require rigorous external validation before clinical use^[50–52]^.

**XAI**: Incorporating interpretability techniques can build clinician trust. Methods like SHAP (Shapley Additive explanations), LIME (Local Interpretable Model-agnostic Explanations), Grad-CAM (Gradient-weighted Class Activation Mapping), and saliency maps can highlight what features (brain regions and cognitive tests) drive a prediction^[46]^. Recent research shows XAI (Grad-CAM, saliency maps, layer-wise relevance propagation, integrated gradients, etc.) can identify AD-relevant biomarkers while providing visual explanations^[46,53–56]^. By explaining model decisions, AI tools can act as transparent decision-support, not opaque replacements. Regulators and clinicians have called for mandated XAI for clinical AI^[28,57]^.

**Clinician engagement and training**: Education programs are needed to improve AI literacy among neurologists, radiologists, and allied staff^[29,33]^. Demonstrating clear benefits (error reduction and faster workflow) can overcome skepticism; indeed, radiologists identified “high expectations of AI’s potential to decrease diagnostic errors and improve care” as facilitators ^[29]^. Involving end-users early in development (human-centered design) ensures the tool meets clinical needs. On-site champions (clinicians who advocate for AI) and multidisciplinary teams (IT, ethics, and clinical) can smooth implementation.

**Regulatory support and guidelines**: Agencies and professional societies must accelerate the development of AI-specific frameworks. For instance, the FDA has published guidance on “predetermined change control” for continually learning devices^[35]^. Clear pathways (e.g., FDA 510(k) categories, CE marking) for AI in neurology should be formalized. The World Health Organization (WHO) and governments can promote standards for data quality and algorithm validation. These policies will give providers confidence in the safety and efficacy of AI diagnostics.

**Infrastructure and integration**: Technical solutions such as cloud-based Saabs (Software as a Service) platforms or on-premises AI servers should be developed for easy integration with PACS/EHR. The RetiSpec–Topcon example shows how embedding AI into existing imaging workflows (Topcon Harmony system) can facilitate adoption^[26]^. Vendors and hospitals should plan for IT support and API connections. Demonstrating cost-effectiveness (e.g., by shorter diagnostic workups) will be key to justify investment. Health systems might pilot AI tools in memory clinics and collect real-world performance data to build the case for wider use.

**Global equity efforts**: To narrow the gap between developed and developing regions, international partnerships are essential (Table 3). HICs can fund and share technology with LMICs. Training programs (online courses and tile-mentoring) can build local expertise in AI and neurology. Low-cost AI solutions tailored for minimal hardware (e.g., smartphone-based cognitive tests) can help resource-limited clinics. Addressing infrastructure (broadband access) is a long-term goal, as is including LMIC data in global AI models. As the WHO emphasizes, “the future of healthcare is digital, and we must do what we can to promote universal access to these innovations and prevent them from becoming another driver of inequity”^[58]^.

**Table 3:** Comparison of AI integration factors in developed versus developing countries

Factor	Developed countries	Developing countries
Data & resources	Extensive research datasets (e.g., AD neuroimaging initiative and UK Biobank) covering diverse biomarkers^[21]^. Strong research funding supports large trials.	Scarce local data on AD; datasets are small and often not publicly available. Research funding for AI is limited.
Infrastructure	Robust digital healthcare infrastructure (integrated EHR/PACS, widespread internet). Ready access to cloud/AI services.	Many regions have intermittent power/internet, few EHR systems. Lack of imaging centers in rural areas.
Regulations & policy	Established medical device regulations (FDA, EMA) and emerging AI guidelines. Insurance/reimbursement pathways evolving.	Regulations for AI and medical devices often absent or outdated; healthcare budgets tight. Out-of-pocket payment common.
Workforce	Larger pool of trained specialists (neurologists, radiologists, and data scientists). Growing AI training programs and startups.	Severe shortage of dementia specialists and AI experts. Brain drain to HICs. Limited exposure of clinicians to AI tools.
Health priorities	Public awareness of dementia: emphasis on preventive and precision medicine. Early adoption of technology in healthcare.	Other health issues (infectious diseases, maternal/child health) compete for resources. Dementia sometimes seen as a low priority.

Despite differences, many solutions are universal: fostering education, standardizing AI protocols, and international collaboration will benefit all settings.

## Limitations

This review has several limitations. First, the literature search was restricted to English-language articles, potentially excluding relevant studies from non-English sources. The studies reviewed primarily focus on research conducted in developed countries, which may limit the applicability of findings to low-income or resource-limited settings. Additionally, the tools discussed are mostly in the early stages of development, making it difficult to generalize their real-world clinical use. The rapid pace of AI advancements may also mean that some of the reviewed technologies are already outdated. Finally, regional differences in healthcare infrastructure may impact the integration of AI tools across various settings.

## Future directions

In the next 3–5 years, key priorities for AI integration in Alzheimer’s diagnosis will include expanding diverse datasets to improve model generalization, enhancing XAI techniques to build clinician trust, and implementing training programs to boost AI literacy among healthcare professionals. Regulatory bodies should expedite the development of clear approval processes, while global collaborations are necessary to ensure equitable access to AI tools, particularly in LMICs. These steps will be crucial for overcoming current barriers and ensuring the widespread adoption of AI in clinical practice.

## Conclusion

AI-based diagnostic tools hold great promise to augment clinical practice in AD by improving accuracy, efficiency, and early detection. A range of promising systems (imaging algorithms and digital tests) is emerging, with some already receiving regulatory approval. However, realizing the potential of AI in AD care will require addressing a host of barriers. Technical issues (limited data and model bias) must be solved through collaboration and sophisticated methods (e.g., federated learning). Equally important are human factors: clinicians need training, trust, and clear evidence before adopting AI recommendations. Regulatory clarity and ethical frameworks will ensure safety and equity. As AI continues to evolve, ongoing validation in diverse clinical populations is essential. By proactively tackling these challenges through transparent research, clinician partnership, and supportive policy, AI diagnostic tools can be integrated responsibly, ultimately improving Alzheimer’s care.

## Data Availability

All data generated during this study are included in this published article.
